# Additional suture augmentation to anterior cruciate ligament reconstruction with hamstring autografts bring no benefits to clinical results, graft maturation and graft-bone interface healing

**DOI:** 10.1186/s12891-024-07426-w

**Published:** 2024-04-17

**Authors:** Qingyang Meng, Ruilan Dai, Cheng Wang, Weili Shi, Yanfang Jiang, Ningjing Liu, Rui Li, Yingfang Ao, Xi Gong, Yong Ma

**Affiliations:** 1 Department of Sports Medicine, Beijing Key Laboratory of Sports Injuries, Peking University Third Hospital, Institute of Sports Medicine of Peking University, 49 North Garden Road, Haidian District, Beijing, 100191 People’s Republic of China; 2 Engineering Research Center of Sports Trauma Treatment Technology and Devices, Ministry of Education, 49 North Garden Road, Haidian District, Beijing, 100191 People’s Republic of China; 3 College of Exercise and health Sciences, Tianjin University of Sport, No.16 Donghai Road, West Tuanbo New Town, Jinghai District, Tianjin, 301617 People’s Republic of China

**Keywords:** Anterior cruciate ligament reconstruction, Suture augmentation, Graft maturation, Graft-bone interface healing

## Abstract

**Background:**

From the perspective of graft protection and early rehabilitation during the maturation and remodeling phases of graft healing, suture augmentation (SA) for anterior cruciate ligament reconstruction (ACLR) has attracted more and more attention.

**Study Design:**

Retrospective study.

**Purpose:**

To determine whether the additional SA affects clinical results, graft maturation and graft-bone interface healing during two years follow-up after ACLR.

**Methods:**

20 ACLRs with additional SA (ACLR-SA group) and 20 ACLRs without additional SA (ACLR group) were performed between January 2020 and December 2021 by the same surgeon and were retrospectively analyzed. Pre- and postoperative International Knee Documentation Committee (IKDC) scores, Lysholm scores, graft failure and reoperation were evaluated. The signal/noise quotient (SNQ) of autografts and the signal intensity of graft-bone interface were analyzed. All 40 patients in ACLR-SA group and ACLR group completed 2-years follow-up.

**Results:**

There was no patient in the two cohorts experienced graft failure and reoperation. The postoperative IKDC and Lysholm scores have been significantly improved compared with preoperative scored in both ACLR-SA group and ACLR group, however, there was no significant difference between two groups. The SNQ of proximal graft of ACLR-SA group (14.78 ± 8.62 vs. 8.1 ± 5.5, *p* = 0.041) was significantly greater while the grades of graft-bone interface healing of posterior tibial was significantly lower than that of ACLR group at 1-year postoperatively (*p* = 0.03), respectively. There were no significant differences between the two groups of the SNQ of proximal, distal medial graft segments, and the graft-bone interface healing grades of anterior femoral, posterior femoral, anterior tibial and posterior tibial at other time points (*p*>0.05).

**Conclusions:**

The additional SA in ACLR had no effect on IKDC scores, Lysholm scores, graft maturation and graft-bone interface healing at 2-year postoperatively. Our research does not support the routine use of SA in ACLR.

## Introduction

Anterior cruciate ligament (ACL) is a critical stabilizing structure in the knee joint that prevents excessive anterior translation of tibia and maintains joint stability. ACL ruptures are common in the physically active population accounting for over 50% of all knee injuries and affecting more than 200,000 people in the United States each year [1]. For patients after ACL rupture, knee-related quality of life is impaired for more than 20 years compared with population norms and peers [2]. Therefore, ACL reconstruction (ACLR) which has proven to be a highly effective technique is deemed necessary to restore native knee kinematics close to the physiological state and to allow patients to return to sports [3].

It is established that fully “maturation” in the intra-articular region of the graft and secure graft-bone interface healing, which are crucial for successful ACLR to facilitate an early and aggressive rehabilitation [4, 5]. However, previous studies have demonstrated that the early graft healing phase was characterized by graft necrosis and hypocellularity without any significant detectable revascularization occurs [6], vigorous activity should not be permitted for patients in the early periods after ACLR [7]. In addition, other studies have found graft tension and stiffness achieved immediately following reconstruction are not maintained postoperatively because of stress relaxation and a temperature increase [8]. In view of the above reasons, some authors added suture tape to the hamstring tendon as an internal brace to provide ACL protection during the healing and remodeling phase, especially in young and active patients to minimize the risk of graft retears [9, 10].

There is limited evidence on the effect of adding suture tape augmentation to the hamstring autografts or allografts following ACLR although this technique has attracted increasing interest and commentary [11–16]. Kaitlin P et al. described the BioBrace, which was a biocomposite scaffold that could both mechanically reinforces the graft while biologically enhancing graft healing [17]. Bodendorfer et al. demonstrated that suture augmentation hamstring ACLRs were associated with improved patient-reported outcomes (PROs), less pain, and a higher percentage of and earlier return to preinjury activity level when compared with standard hamstring ACLRs with minimum 2-years follow-up [9]. Camilo Partezani H et al. also stated that patients that underwent revision ACL reconstruction with a laterally based augmentation procedure recieved a lower failure rate than those who underwent isolated revision ACL reconstruction, and the KT-1000 and pivot-shift examination of them were also significantly better when a lateral augmentation was performed [18]. In another study, ACLR with hamstring autograft and independent suture tape reinforcement was performed safely with low rates of complications, graft failure, and reoperations with similar PROs, function, and return-to-sport rates when compared with hamstring autograft ACLR without suture tape reinforcement at a minimum 2-years follow-up [10]. However, the effect of suture augmentation on hamstring autograft ligamentization and graft-bone interface healing remains unclear, so we conducted this research, and hypothesized that ACLR with suture tape augmentation has similar rate of graft failure and reoperations.

The purposes of this study were to compare (1) PROs, rates of ACLR failure and reoperation, (2) graft ligamentization in the intra-articular region of the graft, and (3) graft-bone interface healing following hamstring autograft ACLR with and without suture augmentation at a 2-years follow-up. We hypothesized that ACLR with suture augmentation has better clinical outcomes and MRI assessment results than ACLR.

## Methods

In a prospective study (trial registration number: NCT04012567; date of first registration: 09/07/2019) which was performed between January 2020 and December 2021 with approval of Medical Science Research Ethics Committee (IRB#: 2019–018), 20 ACLRs with additional SA (ACLR-SA group) and 20 ACLRs without additional SA (ACLR group) were performed by the same surgeon and were retrospectively analyzed. The inclusion criteria were (1) patients of both gender between 18 and 59 years old, (2) with a diagnosis of primary ACL tear confirmed by MRI, Lachman test and anterior drawer test, and (3) the contralateral knee was normal. The exclusion criteria were (1) Patients with open epiphyseal, (2) prior surgery on the affected lower limb, (3) obvious cartilage degeneration, (4) knee flexion degree within 90°, (5) patients who needed to receive simultaneous autologous chondrocyte transplantation. Participants were randomly assigned to two groups using the randomized block group assignment method: ACLR-SA group and ACLR group. Patients of the same surgeon were assigned within the same block group, the number of each block group was 20, and then the 20 patients within that block group are randomly assigned to groups. All patients were blinded to the treatment and received ACLR with the similar surgical technique and pre- and postoperative rehabilitation.

Pre- and postoperative PROs (International Knee Documentation Committee (IKDC) scores and Lysholm scores) and clinical outcomes (Lachman test and anterior drawer test) were evaluated by the same research doctor. The MRI imaging analysis of graft ligamentization in the intraarticular region of the graft and graft-bone interface healing was done by the other doctor. Both doctors above were blinded to the surgical technique whether suture tape was added. ACLR failure was defined as graft rupture confirmed by MRI, Lachman test and anterior drawer test.

### ACLR technique

Single-bundle ACLRs with hamstring autografts (gracilis and semitendinosus tendon) were performed as previously described [19]. Briefly, the central point of I.D.E.A.L femoral tunnel [20] was located with a 5.5-mm femoral guide (DePuy Mitek, Raynham, MA, USA) using the apex of the deep cartilage (ADC) [21] as the landmark at 120° of knee flexion through the anteromedial portal. The tibial tunnel was located at the anatomical central portion of the remnant ACL using an Acufex tip-to-tip drilling guide (Smith and Nephew, Andover, MA, USA) set at an angle of 55°. Both the tibial and femoral bone tunnels were drilled according to the graft diameter (7–8 mm). An Endobutton (Smith and Nephew Endoscopy, Andover, MA) suspensory system was flipped to establish femoral fixation of hamstring autografts harvested from the ipsilateral leg. For ACLR-SA group, the nonabsorbable ultra-high molecular weight polyethylene/polyester suture tape (Arthrex, Naples, FL, USA) was passed through the loop of Endobutton suspensory system with hamstring autografts. Tibial fixation was performed at 0° of knee flexion via bioresorbable interference screw (Smith and Nephew, Andover, MA, USA). Finally, the suture tails of sutured grafts were secured with a staple made of Kuntscher wire in ACLR group and Knotless Anchors (SwiveLock; Arthrex, Naples, FL, USA) in ACLR-SA group, respectively.

### Rehabilitation

All patients followed the same rehabilitation protocol with the permission of immediate weight-bearing. A functional brace was used to maintain the knee that underwent ACLR or ACLR-SA in a fully extended position within the first two weeks postoperatively. Then, range-of-motion exercises were performed gradually with the goals of achieving 90° knee flexion within two weeks and 120° knee flexion within three months postoperatively. Straight-leg raises and isometric quadriceps contractions were performed immediately after surgery. All patients underwent home-based rehabilitation after discharge with the goals of recovering exercises of daily living level within three months, participating gradually in moderate sports activities six months and returning to competitive sports ten months after operation with muscle strength recovering at least 90% compared with the contralateral leg.

### MRI imaging analysis

**Fig. 1:**
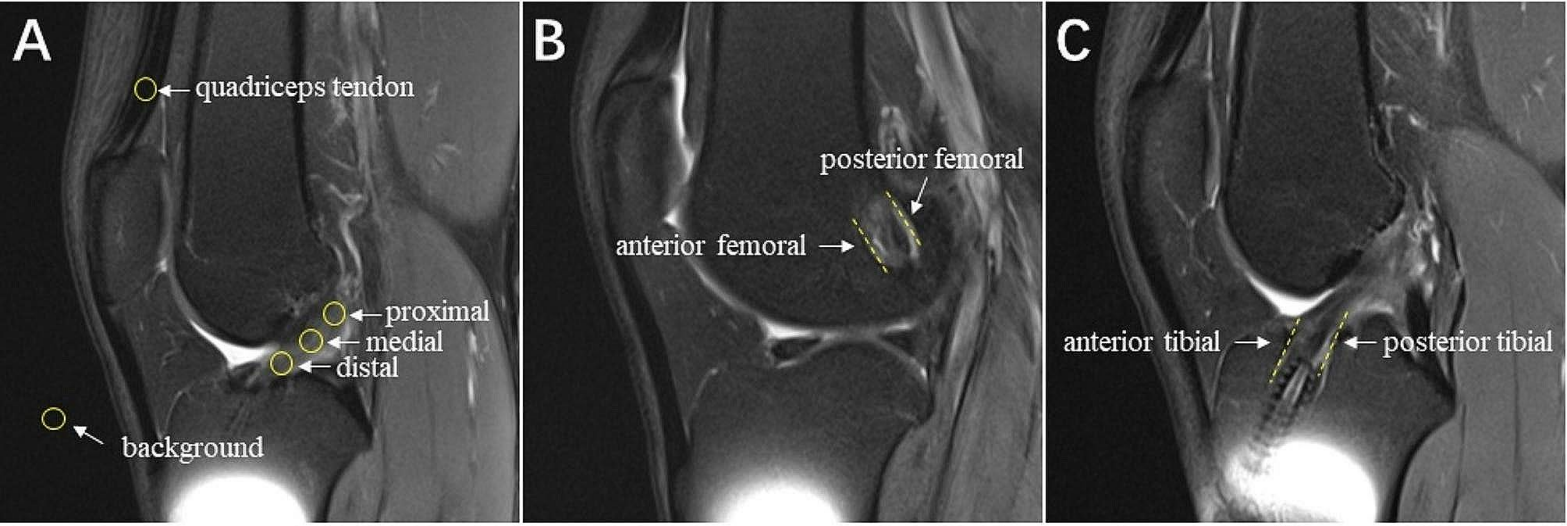
The evaluation of autograft maturation and graft-bone interface healing on MRI images. **A**. The SI of hamstring autograft (yellow circle) was obtained on the proximal, medial and distal segments of graft. SNQ = (SI of hamstring autograft - SI of quadriceps tendon) / SI of background. **B** and **C**. The anterior and posterior SI of graft-bone interface at femoral tunnel and 1/3 proximal tibial tunnel (yellow imaginary line) was obtained and classified into 4 grades. SI: signal intensity, SNQ: signal/noise quotient

The MRI examinations for graft maturation and graft-bone interface healing were performed in a relaxed extended position with a 3.0-T MRI scanner (MAGNETOM Verio, A Tim system, Siemens, Germany) at 6 months, 1 year and 2 years after surgery. The imaging protocol was standardized and similar in both groups. The intra-articular region of the hamstring autograft was divided into three segments: proximal, medial and distal based on T2-weighted oblique axial images. The signal intensity (SI) of hamstring autograft was obtained using a 11.88 mm^2^ circle regions of interest on the different segment of graft via Centricity RIS/PACS CE software (GE, USA). The signal/noise quotient (SNQ) of each graft segment was calculated using the following equation: SNQ = (SI of hamstring autograft - SI of quadriceps tendon) / SI of background (Fig. 1A) [22]. The graft-bone interface healing characterized by fibrous interzone between the autograft and the bone tunnel wall was also evaluated based on T2-weighted oblique axial images. The SI of graft-bone interface was classified into 4 grades based on it was similar to that of the patellar tendon (Grade 3), similar to that of skeletal muscle (Grade 2), greater than that of muscle but less than that of joint fluid (Grade 1) and similar to that of joint fluid (Grade 0) [23]. On the first slices in which the femoral tunnel and proximal tibial tunnel appears intact, respectively, both the anterior and posterior SI of graft-bone interface at femoral tunnel and 1/3 proximal tibial tunnel was obtained (Fig. 1B and C). Two surgeons with more than ten years’ working experience carried out the measurement, and each of them repeated the measurement three times.

### Statistical analysis

Statistical analysis was performed using SPSS software Version 25 (IBM, Chicago, USA). Kolmogorov–Smirnov test was used to evaluate whether the data conformed to a normal distribution. Continuous data was described as mean ± standard deviation (SD), whereas non-normal distribution data are expressed as the mean with interquartile range, M (Q1, Q3). Categorical data was described as number, and the chi-square test was used to analyze the distribution the graft-bone interface healing grades of anterior femoral, posterior femoral, anterior tibial and posterior tibial. Paired samples T test was used for intra-group comparison, independent samples T test was used for inter-group comparison, and Mann-Whitney tests was used for non-normal data. A p-value of < 0.05 was considered statistically significant.

## Results

All 40 patients in ACLR-SA group and ACLR group completed 2-years follow-up. Demographic characteristics of the ACL tear participants are described in Table 1. There were no significant differences between the two groups in sex, age, laterality, height, weight and body mass index (BMI) (*p*>0.05).

**Table 1:** Demographic data of the ACL tear participants in the study groups

Characteristics	ACLR-SA group (*n* = 20)	ACLR group (*n* = 20)	p value
Gender (male/female)	15/5	15/5	1
Age (years)	33. 7 ± 8.8	36.4 ± 11.0	0.396
Laterality (left/right)	11/9	6/14	0.115
Height (cm)	175.7 ± 8.3	173.5 ± 7.7	0.762
Weight (Kg)	78.8 ± 14.7	76.2 ± 14.7	0.698
BMI (Kg/m^2^)	25.4 ± 3.5	25.2 ± 3.8	0.989

ACL, anterior cruciate ligament; ACLR, anterior cruciate ligament reconstruction; SA, suture augmentation; BMI, body mass index

There was no patient in the two cohorts experienced reoperation and graft failure confirmed by MRI, Lachman test and anterior drawer test during the 2-years follow-up period. PROs data was presented in Table 2. IKDC scores and Lysholm scores of ACLR group were significantly higher than that of preoperative PROs (*p*<0.05) but have no significant difference between ACLR group and ACLR-SA group (*p*>0.05).

**Table 2:** Patient-Reported Outcomes (PROs)

	ACLR-SA group (*n* = 20)	ACLR group (*n* = 20)	p value
Preoperative PROs			
IKDC scores	60.3 ± 6.5	63.3 ± 7.0	0.329
Lysholm scores	78.3 ± 7.8	80.9 ± 9.8	0.573
6 months postoperative PROs			
IKDC scores	67.1 ± 6.0	67.8 ± 5.1	0.812
Lysholm scores	80.0 ± 13.8	82.3 ± 14.4	0.066
1 year postoperative PROs			
IKDC scores	75.4 ± 5.9	76.3 ± 6.2	0.563
Lysholm scores	92.8 ± 6.8	89.1 ± 8.62	0.052
2 years postoperative PROs			
IKDC scores	74.3 ± 5.8	74.3 ± 6.0	0.237
Lysholm scores	88.4 ± 9.5	93.4 ± 8.5	0.110

ACLR, anterior cruciate ligament reconstruction; SA, suture augmentation; IKDC, International Knee Documentation Committee

The postoperative SNQ data of intra-articular hamstring autograft by MRI assessment was presented in Table 3. The SNQ of proximal segment in ACLR-SA group was significantly greater than that of ACLR group at 1-year postoperatively (*p* = 0.041). There were no significant differences between the two groups of the SNQ of proximal, distal and medial segments at other time points (*p*>0.05).

**Table 3:** SNQ of intra-articular hamstring autograft by MRI assessment

	ACLR-SA group (*n* = 20)	ACLR group (*n* = 20)	p value
6 months postoperative SNQ			
proximal	13.4 ± 5.7	10.4 ± 7.5	0.178
medial	14.7 ± 6.8	12.9 ± 9.2	0.462
distal	15.8 ± 8.4	11.9 ± 8.8	0.156
1-year postoperative SNQ			
proximal	14.78 ± 8.62	8.1 ± 5.5	**0.041**
medial	15.75 ± 8.67	10.9 ± 5.7	0.191
distal	16.10 ± 8.62	14.2 ± 7.9	0.752
2 years postoperative SNQ			
proximal	11.9 ± 6.3	7.7 ± 5.5	0.097
medial	11.5 ± 6.0	9.1 ± 4.7	0.136
distal	11.4 ± 4.3	10.8 ± 4.8	0.571

SNQ, signal/noise quotient; MRI, magnetic resonance imaging; ACLR, anterior cruciate ligament reconstruction; SA, suture augmentation

The grades of graft-bone interface healing between the two groups were demonstrated in Table 4. The distribution of 3 grades of graft-bone interface healing of posterior tibial in ACLR-SA group was significantly lower than that of ACLR group at 1-year postoperatively (10% vs. 45%, *p* = 0.030). There were no significant differences between the two groups of other segments grades of graft-bone interface healing at other time points (*p*>0.05). The total sample of 40 subjects achieves 56% power to detect differences among the means versus the alternative of equal means using a Rank Sum test with a 0.05 significance level.

**Table 4:** Grades of graft-bone interface healing by MRI assessment

	ACLR-SA group (n) Grade 0/1/2/3	ACLR group (n) Grade 0/1/2/3	p value
6 months postoperative			
Anterior femoral	1/5/10/4	0/7/10/3	0.834
Posterior femoral	2/6/9/3	0/6/8/6	0.463
Anterior tibial	1/5/9/5	0/6/6/8	0.612
Posterior tibial	1/4/8/7	0/6/6/8	0.781
1 year Postoperative			
Anterior femoral	0/3/12/5	0/0/17/3	0.131
Posterior femoral	0/3/11/6	0/1/16/3	0.281
Anterior tibial	0/2/15/3	1/2/10/7	0.264
Posterior tibial	0/4/14/2	1/3/7/9	**0.030**
2 years Postoperative			
Anterior femoral	0/0/8/12	0/0/12/8	0.343
Posterior femoral	0/0/13/7	0/0/11/9	0.748
Anterior tibial	0/0/11/9	0/0/9/11	0.752
Posterior tibial	0/0/9/11	0/0/10/10	1

MRI, magnetic resonance imaging; ACLR, anterior cruciate ligament reconstruction; SA, suture augmentation

## Discussion

The most important finding of this study was that ACLR with or without suture augmentation had similar PROs, graft maturation and graft-bone interface healing with 2-year follow-up. These results suggested that additional suture augmentation to the hamstring autografts did not improve clinical results, graft maturation and graft-bone interface healing for ACLR. Although only 40 subjects were finally included, the power results showed that the sample size of 40 was acceptable. Therefore, our research hypothesis was not valid.

From the perspective of graft protection against irreversible lengthening and early rehabilitation during the maturation and remodeling phases of healing, suture tape augmentation or reinforcement for ACLR has attracted more and more attention. Biomechanical studies have shown that independent reinforcement of soft-tissue grafts with suture tape leads to significantly reduced elongation and higher ultimate failure load according to in vivo native ACL function data without stress-shielding the soft tissue graft [12], significantly improves dynamic elongation at increased stiffness and ultimate strength on the performance especially of tripled smaller-diameter grafts for ACLR with tibial screw fixation [15]. These results provide biomechanical evidence for adding suture tapes to the grafts in ACLR.

Clinical studies are scarce and larger clinical trials will have to prove whether this small addition to grafts will have a positive impact on ACLR results [13, 14]. Allom et al. reported that the addition of suture tape to an autologous hamstring graft construct did not reduce instrumented sagittal knee laxity in the first 6 months after ACLR [11]. Parkes et al. demonstrated that ACLR with independent suture tape reinforcement was associated with lower rates of complications, graft failure and reoperations compared with ACLR without independent suture tape reinforcement, while the PROs, function, and return-to-sport rates were similar [10]. One study reported suture augmentation/ reinforcement ACLRs were associated with improved PROs, less pain, and a higher percentage of and earlier return to preinjury activity level when compared with standard hamstring ACLRs with minimum 2-years follow-up [9], and for those who less than 25 years old that ACL revision, maybe it is more appropriate to make surgical indications for ACL reconstruction combined with extra-articular lateral tenodesis [24]. However, Alan M J et al. demonstrated that a single-bundle, hamstring ACLR in combination with a lateral extra-articular tenodesis (LET) reduced the risk of ACLR failure in young [25]. Overall, the literature reported similar or better clinical outcomes with ACLR-SA than with ACLR. Our results showed that ACLR group had similar effect on 6 months, 1-year postoperative, 2-years postoperative IKDC scores and Lysholm scores, however, the SNQ of proximal graft and Grades of graft-bone interface healing of posterior tibial of ACLR-SA group was significantly greater than that of ACLR group at 1-year postoperatively, suggesting that additional suture tape augmentation to ACLR did not improve graft-bone interface healing and have negative effects on PROs and graft ligamentization.

After a thorough literature search, we confirm that our study was the first to investigate the effect of additional suture tape on graft maturation and graft-bone interface healing in ACLR. According to the results of our study, the additional suture augmentation in ACLR had no different effect on IKDC scores compared with standard ACLR. According to our study, the SNQ of proximal intra-articular graft in ACLR-SA group was significantly greater than that of ACLR group at 1-year postoperatively, whereas the SNQ of the other segments did not differ significantly between the ACLR-SA and ACLR groups at 6 months,1 year and 2 years postoperatively. It has been shown that lower SNQ of graft is associated with better graft maturation after ACLR [26]. Lutz et al. found that the ACL graft signals approximated the signal of a native intact ACL at 12 and 24 months; however, the autograft maturation on sequential postoperative MRI is not correlated with clinical outcome and anterior knee stability [27]. The effect of suture tape on graft maturation needs to be studied on a larger scale and with longer follow-up time in the future.

The secure graft-bone interface healing may reduce the risk of graft failure and enable early aggressive rehabilitation [23]. The graft-bone interface healing process is generally divided into four stages: inflammation, proliferation, matrix synthesis and matrix remodeling [28]. To evaluate the effect of suture tape on the anterior and posterior graft-bone interface healing, the SI of graft-bone interface at femoral tunnel and 1/3 proximal tibial tunnel was measured on the first slice in which the femoral tunnel appears intact and the tibial tunnel showing complete, respectively. At 1-year postoperatively, y, there were more Grade 3 posterior tibial graft-bone interface healing patients in ACLR group (2/20, 10%) than the ACLR-SA group (9/20, 45%), indicating the additional suture tape to hamstring autograft also had a possible negative effect on graft-bone interface healing.

Overall, the most important finding of this study is that the additional suture tape to hamstring autograft had no effect on graft-bone interface healing. Stress-shielding may be responsible for this. In a rabbit model study, stress-shielding contributed unfavorable influence on graft maturation not only in the mid-substance but also at the ligament-bone junction [29]. Therefore, stress-shielding must be considered when adding suture tape to hamstring autograft in ACLR [30]. It is unclear if or when the suture tape will break. More clinical and biomechanical evidence of the effect of additional suture tape on graft maturation in the intra-articular region of the graft and graft-bone interface healing are needed.

### Limitations

The present study has some limitations. Firstly, larger scale of participants and further follow-up is necessary in the future because the total sample size in our study was small and the follow-up period was only 2 years. Secondly, IKDC scores, Lysholm scores and graft failure rates were compared to evaluate the clinical results of ACLR with or without SA, while Tegner activity, rates of return to sport and complications were not collected. Finally, we did not analyze the effect of concurrent diseases such as meniscus injury and cartilage damage on the above results.

## Conclusions

The additional suture augmentation in ACLR had no effect on IKDC scores, Lysholm scores, graft maturation and graft-bone interface healing at 2-year postoperatively. Our research does not support the routine use of suture augmentation in ACLR.

## Data Availability

All data generated or analyzed during this study are included in this manuscript.
